# Workers’ Compensation Status: Does It Affect Orthopaedic Surgery Outcomes? A Meta-Analysis

**DOI:** 10.1371/journal.pone.0050251

**Published:** 2012-12-05

**Authors:** Vinícius Ynoe de Moraes, Katelyn Godin, Marcel Jun Sugawara Tamaoki, Flávio Faloppa, Mohit Bhandari, João Carlos Belloti

**Affiliations:** 1 Division of Hand and Upper Limb Surgery, Department of Orthopaedics and Trauma, Universidade Federal de São Paulo, São Paulo, Brazil; 2 Division of Orthopaedic Surgery, Department of Surgery, McMaster University, Hamilton, Ontario, Canada; Harvard Medical School, United States of America

## Abstract

**Introduction:**

Previous reviews have demonstrated that patient outcomes following orthopaedic surgery are strongly influenced by the presence of Workers’ Compensation. However, the variability in the reviews’ methodology may have inflated the estimated strength of this association. The main objective of this meta-analysis is to evaluate the influence of Workers’ Compensation on the outcomes of orthopaedic surgical procedures.

**Methods:**

We conducted a systematic search of the literature published in this area from 1992–2012, with no language restrictions. The following databases were used MEDLINE (Ovid), Embase (Ovid), CINAHL, Google Scholar, LILACS and Pubmed. We also hand-searched the reference sections of all selected papers. We included all prospective studies evaluating the effect of compensation status on outcomes in adult patients who had undergone surgery due to orthopaedic conditions or diseases. Outcomes of interest included disease specific, region specific and/or overall quality of life scales/questionnaires and surgeons’ personal judgment of the results. We used an assessment tool to appraise the quality of all included studies. We used Review Manager to create forest plots to summarize study data and funnel plots for the assessment of publication bias.

**Results:**

Twenty studies met our eligibility criteria. The overall risk ratio for experiencing an unsatisfactory result after orthopaedic surgery for patients with compensation compared to non-compensated patients is 2.08 (95% CI 1.54–2.82). A similar association was shown for continuous data extracted from the studies using assessment scales or questionnaires (Standard Mean Difference = −0.70 95% CI -0.97- −0.43).

**Conclusions:**

Among patients who undergo orthopaedic surgical procedures, those receiving Workers’ Compensation experience a two-fold greater risk of a negative outcome. Our findings show a considerably lower estimate of risk compared to previous reviews that include retrospective data. Further research is warranted to determine the etiological explanation for the influence of compensation status on patient outcomes.

**Systematic Review Registration Number:**

CRD42012002121

## Introduction

The success of a surgical intervention in orthopaedic medicine is influenced by several factors including the appropriateness of the surgical indication and the surgeon’s skill level and experience with the specific procedure. A patient’s compensation status may also influence how a patient fares following orthopaedic surgery. In clinical practice, orthopaedic surgeons often treat patients who are receiving Workers’ Compensation benefits for their injuries and/or conditions [1]–[4]. Several studies demonstrate that receiving Workers’ Compensation may correlate to a negative prognosis following treatment for a vast range of health conditions [5]–[11]. It is important to understand this phenomenon and to encourage orthopaedic surgeons to consider patients’ compensation status when assessing their expected outcomes[12]–[14].

Surgeons should utilize the best available studies as a guide when attempting to assess the influence of compensation status on patients’ outcomes following orthopaedic surgery. Despite the well-established merit of randomized controlled trials in evidence-based medicine, incorporating randomization and blinding into a study’s design is wrought with challenges and often is not feasible or ethical in the field of orthopaedic surgery[15]–[17]. Therefore, the results from high-quality observational studies are often the best source of evidence to be considered in clinical decision-making within orthopaedic medicine[16]–[19]. This recognition that observational studies have a critical place within evidence-based medicine is supported extensively in the literature [16], [17], [20], [21].

The influence that compensation status has on the prognosis of patients undergoing surgical treatment for musculoskeletal disorders has been investigated by a number of clinical studies [2], [7], [22], [23] and systematic or meta-analytic reviews[24]–[27]. However, there are methodological flaws in the design of many of these studies which diminishes the confidence one may have in their findings. For example, two of the systematic reviews [24], [27] considered only disease-specific studies and were therefore limited to a very small number of studies. This approach leads to results that lack generalizability across patient populations. Another review on this topic featured a very broad eligibility criteria and included retrospective studies as well as studies that were conducted in the 1960s. We feel that this may had lead to an imprecise magnitude of the present-day relationship between compensation status and patient outcomes as the working world has undergone substantial changes in the past several decades. One study reported a 4.72 odds ratio, showing that patient outcomes are negatively influenced by Worker’s compensation [26]. From our experience in clinical practice, this finding seems somewhat unrealistic and serves as an illustrative example of the possible inflated estimates from existing clinical studies and systematic and meta-analytic reviews.

As such, no best-evidence approach is currently available in the literature to effectively demonstrate the magnitude of the influence of the compensation status on patient outcomes following orthopaedic surgery. We feel this is valuable information for practicing surgeons who encounters this type of patient population in their practice. We conducted a systematic review and meta-analysis to resolve this issue and to obtain evidence with a greater confidence and power due to the synthesis of the results of primary studies [28], [29].

The objective of this systematic review and meta-analysis is threefold, to assess: 1) the general influence of compensation status on patient outcomes through inclusion of only high quality prospective studies focusing on adult patients who undergo surgery for a variety of musculoskeletal orthopaedic conditions (both traumatic and non-traumatic); 2) if this relationship varies between traumatic and non-traumatic injuries; and 3) if outcomes vary for well-known Workers' Compensation surgical populations: upper extremity non-traumatic surgery (e.g. rotator cuff tears and nerve compression syndromes) and spine surgery (primarily degenerative disk diseases).

## Materials and Methods

This meta-analysis follows the PRISMA Statement [30] reporting recommendations. This review’s protocol can be found in the PROSPERO database (http://www.crd.york.ac.uk/prospero/) under the registration number CRD42012002121.

### Search Strategy

A literature search was conducted with the assistance of a third investigator not related to the study. We utilized the following databases: MEDLINE (Ovid), Embase (Ovid), CINAHL, Google Scholar, LILACS and the Cochrane Library. Following this initial review, we hand-search the references sections of papers we had included at this stage in order to locate additional studies and to avoid missing relevant papers. We did not exclude any studies on the basis of language. We included papers published between 1992 and 2012 (May, 02). Our search strategy is shown in **Appendix S1**.

### Eligibility Criteria

**Figure 1:**
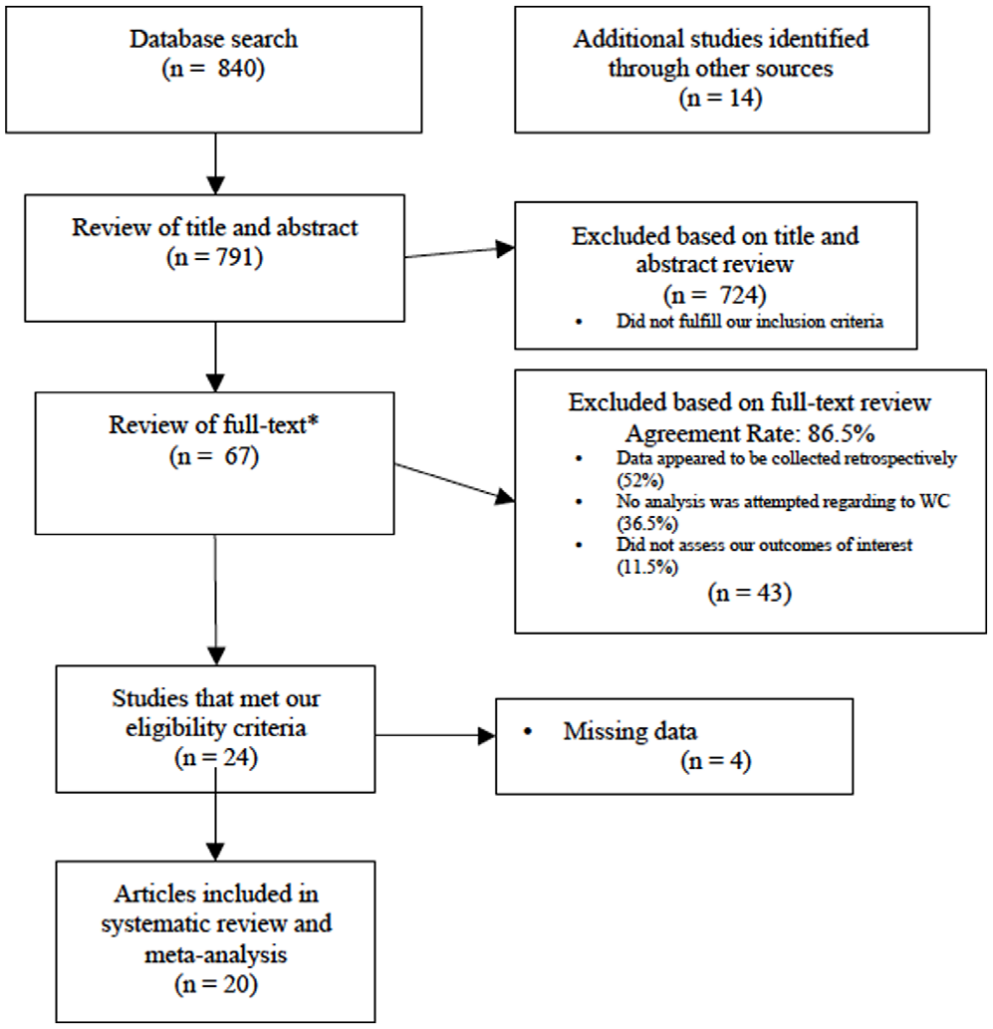
Flow Chart of Study Process. This figure demonstrates the various stages of our systematic review and depicts the reasons why certain papers were excluded.

Studies were included if they met the following criteria: 1) The data was collected and analyzed prospectively; 2) The authors assessed the influence of compensation status specifically; 3) Orthopaedic surgery was the main intervention; and 4) The study was published in the last 20 years, between 1992 and 2012. Also, to be included, the study had to report at least one of the following outcomes: region or disease-specific scales or questionnaires (e.g. the Disabilities of the Arm, Shoulder and Hand measure [32] or Oswestry Disability Index [33]); quality of life assessment scales (e.g. the SF-36); pain assessment scales (e.g. through use of a Visual Analogical Scale), study-specific developed grading criteria for assessing the outcomes. We excluded studies that met any of the following criteria: 1) The study involved non-surgical treatments; 2) Patient data was collected retrospectively; and 3) The study did not report any of the outcomes of interest, as described above. We included studies after a 2-stage assessment, as depicted in the study flow chart (**Figure 1**). Disagreement regarding which studies should be included were resolved by group discussion (VY, KG). We foresaw a third part consultation (JB, MB) if consensus was not reached.

### Data Management: Collection and Extraction

Both qualitative and quantitative data were extracted following full text analysis of the included studies. We also extracted additional information on the studies’ design, funding, population under investigation, intervention, control (if applicable), outcome, duration of follow-up, criteria for Workers’ Compensation identification and the population’s working demands. We also grouped included studies by the condition that was treated in the patient population as follows: 1) Non-traumatic spine surgery (e.g. disc diseases and fusion surgery); 2) Upper limb surgery (e.g. rotator cuff tear repairs, carpal tunnel release surgery); and 3) Fractures. We also grouped studies based on the patients’ cause of injury or condition (traumatic versus non-traumatic). If authors included populations from both traumatic and non-traumatic cases, we decided to group the study based on the authors’ most reported case. We grouped the studies in these ways in order to perform valuable subgroup analyses.

We collected both continuous and dichotomous data from the included studies and analyses were performed in individual forest plots. Many of studies reported several outcomes using different scales and measures for assessment of their patient population. Whenever possible, we focused on one dichotomous and one continuous outcome per study. In the event that a study reported more than one dichotomous or continuous outcome, we extracted data preferentially based on the type of measure. We extracted data based on the following order: 1) Authors’ categorizations of outcomes, based on region or disease-specific tools (e.g. patient scores <80 = unsatisfactory outcome; >80 = satisfactory outcome); 2) Authors’ judgment of the outcome, based on quality of life scales or questionnaires; 3) Authors’ judgment of the outcome, based on pain measurements; 4) Authors’ judgment of the outcome, based in his/her subjective clinical appraisal. We categorized data from poor, fair or unsatisfactory groups as unsatisfactory outcomes. Dichotomous data from items 1 to 4 were collected as the number of unsatisfactory results from Workers’ Compensation patients versus patients receiving no compensation. When the results were reported in a non-categorized manner, we obtained scores from studies that reported continuous outcomes and then plotted them in a meta-analysis. We did not categorize the patients’ outcomes based on our personal judgments, since we believed this may introduce bias.

Continuous data was collected from reported means, standard deviations, and the number of patients in each group. When study data was missing and/or unclear in the published paper, we attempted to contact the authors by email to clarify or provide us with additional data from their study. After it was entered, all data was verified by two authors (VY, JB).

### Study Quality Assessment

Two authors (VY, KG) assessed the quality of all included studies by a tool developed specifically to appraise the risk of bias within observational studies [34]. This tool contains objective questions that appraise the risk of bias in eight areas: selection bias, confidence in the assessment of the exposure, confidence in the recognition of the outcome, matching or statistical adjustments for comparisons, confidence in the assessment of the outcome, confidence in the assessment of the presence or absence of prognostic factors, adequateness of the follow-up period and similarities between co-interventions. All the eight aspects were graded in a four-category scale from the highest to the lowest risk of bias. The tool can be seen in **Appendix S2**.

### Statistical Analysis

A priori, we decided to analyze data according to the following groups: 1) A comprehensive analysis, with the inclusion of all studies; 2) A subgroup analysis of traumatic versus non-traumatic conditions; and 3) Additional subgroup analyses for each of the following: spine, upper limb non-traumatic conditions and fractures. An overall and subgroup meta-analysis was performed for all subgroups if there were at least three studies available for pooling.

We utilized Review Manager [35] (version 5.1) to conduct the meta-analysis. Since we sought to investigate both dichotomous and continuous variables, we used two different approaches. All calculations for dichotomous data were performed using a random-effects model [36] and Mantel-Haenszel method. Continuous data was calculated using an inverse variance method and also in a random-effects model. We provided measures as risk ratios using a 95% confidence intervals. We demonstrated a sum of the risk or mean difference from the studies where this was possible. For dichotomous variables, comparisons between the subgroups were performed using a chi-square test, within Review Manager software.

Mean differences and 95% confidence intervals were also summarized in forest plots. Heterogeneity was assessed by I^2^ statistics. We analyzed publication bias by funnel plots.

## Results

Of the 805 references screened by title and abstract, sixty-seven were selected for full text assessment. From these, 20 fulfilled the eligibility criteria and were included in the meta-analysis[37]–[56]. The included studies contain data from 2608 patients, all of whom underwent orthopaedic surgical procedures. Four studies [39], [47], [48], [50] provided continuous and dichotomous data and were included in dichotomous and continuous data pooling. **Figure 1** provides a pictorial representation of the stages of the review process.

The majority of the studies (14/20, 70%) included in our review were conducted in the United States[37]–[39], [41], [43], [44], [46], [48], [49], [51]–[56], and almost three-quarters of the studies report the results of surgical outcomes for one of three important Workers’ Compensation populations: lumbar spine injuries [38], [39], [43], [45], [49], rotator cuff diseases [40], [46], [47], [50], [52], [54] and carpal or ulnar tunnel syndrome [44], [51], [55]. The characteristics of included studies are detailed in **Table 1**.

**Table 1:** Characteristics of Included Studies.

Characteristic	N (%)
*Origin of Study*	
United States	14 (70)
Canada	4 (20)
Europe	2 (10)
*Study Design*	
Prospective case series	16 (80)
Randomized controlled trials	4 (20)
Study Designed to Assess Influence of CompensationStatus	
Yes	5 (25)
*Surgical Intervention*	
Lumbar spine discectomy, with or without fusion	5 (25)
Rotator cuff repair, with or without acromioplasty	6 (30)
Carpal or cubital tunnel release	3 (15)
Knee reconstruction	2 (10)
Other	4 (20)
*Number of Surgeons*	
1	3 (15)
2	9 (25)
>2	8(40)
*Mean Follow-up*	
<6 months	0 (0)
6–24 months	10 (50)
>24 months	10 (50)
*Method of Outcome Assessment*	
Region or disease-specific or quality of lifeinstrument/scale	12 (60)
Pain instrument/scale	4 (20)
Patient self-reported satisfaction	2 (10)
Surgeon’s subjective appraisal	2 (10)
*Gender* *	**Mean (Range)**
% male patients	58 (35–83)
% of Patients Lost to Follow-up**	13.9(0–28.7)
# of Patients*	129.3(16–539)
Mean Age of Participants***	28–56 (Range)

* Data available from 16 studies.

** Data available from 10 studies.

*** Data available from 15 studies.

**Figure 2:**
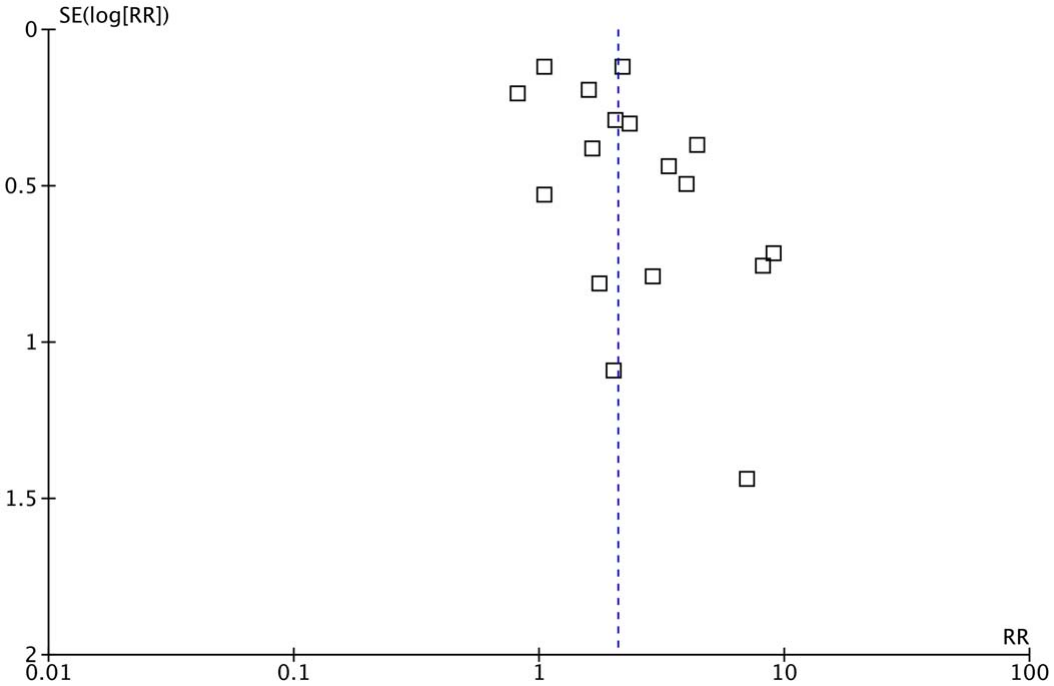
Funnel Plot for Publication Bias. This funnel plot was used to assess whether publication bias was potentially present in our meta-analysis.

Following an assessment of the studies’ quality, six (30%) of the twenty studies were considered to be of low quality [43], [44], [48], [49], [51], [55]. **Table 2** **and** **3** displays the findings from the study quality appraisal. Assessment of the funnel plot demonstrates that publication bias was unlikely a considerable factor (**Figure 2**).

**Table 2:** Quality Assessment.

Study/Quality Scale	Q1	Q2	Q3	Q4	Q5	Q6	Q7	Q8	Overall Rating
Antoniou, 2000	I	I	II	II	II	II	II	I	***
Asch, 2002	II	II	II	III	II	II	I	III	**
Atlas, 2009	III	II	II	II	II	II	II	III	**
Balyk, 2008	II	II	I	II	II	I	III	II	***
Barrett, 2001	II	II	II	III	III	II	II	I	**
Buckley, 2002	III	II	II	III	III	II	II	II	**
Deustsch, 2006	II	II	II	IIII	III	III	III	III	*
Glowacki, 1997	III	II	II	IIII	III	IIII	III	II	*
Greenough, 1994	II	II	II	III	II	III	II	II	**
Henn III, 2008	III	II	II	I	I	II	II	II	***
Johannsen, 1997	II	II	II	II	III	II	II	II	**
Lin, 2000	II	II	II	III	III	III	II	II	*
MacKay, 1995	II	II	II	III	III	IIII	III	III	*
McKee, 2000	II	II	II	II	III	II	III	II	**
Nagle, 1994	II	II	II	III	IIII	III	III	II	*
Nicholson, 2003	II	II	II	II	II	II	II	I	***
Rosenberger, 2008	I	II	II	III	II	II	II	I	***
Spangehl, 2002	I	II	II	II	II	I	III	II	***
Straub, 1999	III	II	II	IIII	III	III	II	IIII	*
Westkaemper, 1998	II	II	I	IIII	II	II	II	II	**

**Table 3:** Quality Assessment Ratings.

Scores	Ratings
*I*	Very Low Risk of Bias
*II*	Low Risk of Bias
*III*	High Risk of bias
*IIII*	Very High Risk of Bias
*	Low Quality
**	Moderate Quality
***	High Quality

**Figure 3:**
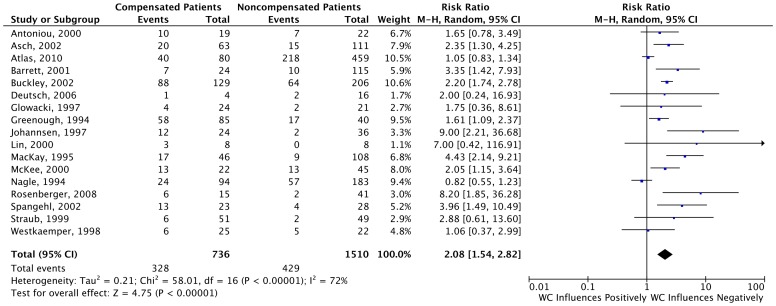
Forest Plots for Studies Reporting Dichotomous Data. This forest plot depicts the results of the 17 studies that reported dichotomous data.

**Figure 4:**
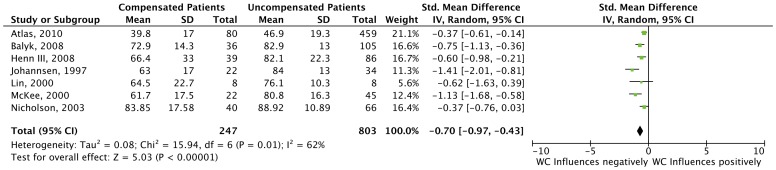
Forest Plots for Studies Reporting Continuous Data. This forest plot depicts the results of the 7 studies that reported continuous data.

In the overall analysis of all studies reporting dichotomous outcomes (n = 17), our results demonstrated that Workers’ Compensation patients have worse outcomes when compared to non-compensated patients (RR = 2.08, 95% CI 1.54–2.82), as shown in **Figure 3**. The overall comparison of the seven studies that reported only continuous data from the scales or questionnaires showed the same trend, (Standard Mean Difference = −0.70 95% CI -0.97- −0.43). The results of this are shown in **Figure 4**.

**Figure 5:**
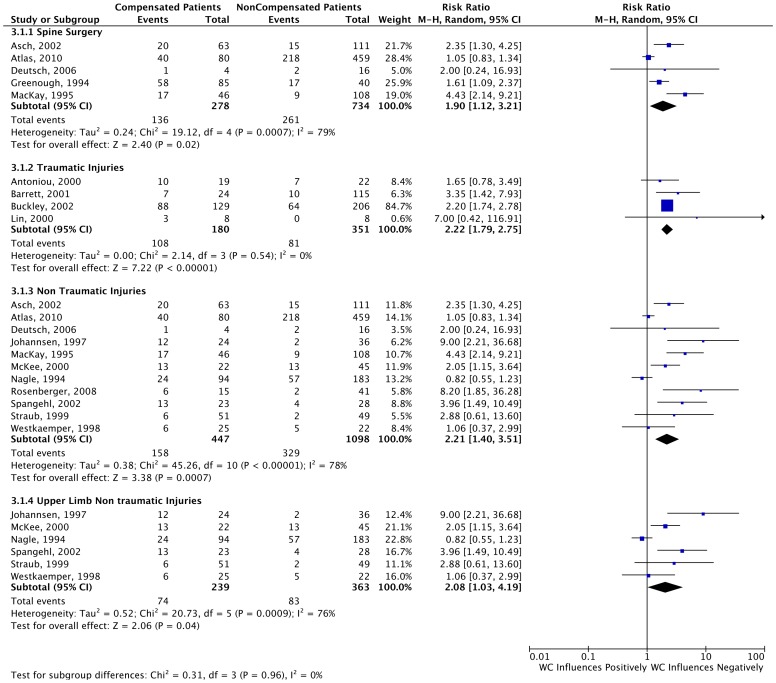
Forest Plots for Studies Reporting Dichotomous Data. This forest plot depicts the results of the subgroups that we decided to analyze a priori.

We further examined if the relationship between compensation status and outcome varied depending on the condition/disease being treated. The results of our subgroup analyses are as follows: 1) lumbar spine injuries, five studies (RR = 1.90 95% CI 1.12–3.21); 2) upper limb injuries, six studies (RR = 2.08 95% CI 1.03–4.19); 3) traumatic injuries [37], [41], [42], [48], four studies (RR = 2.22 95% CI 1.79–2.75); and 4) non-traumatic injuries[38]–[40], [43]–[47], [49]–[56], eleven studies (RR = 2.21 95% CI 1.40–3.51). We found no differences between these subgroups (p = 0.96). These subgroup analyses are depicted in **Figure 5**.

We also performed further explorative analyses that were not initially set in our protocol. The additional subgroups were 1) grouped by studies’ country of origin; 2) studies that specially assessed Workers’ Compensation; and 3) only high-quality studies. Again, the results from these analyses reflected the same findings as seen in our overall comparison. We provide the sum of the risks from these studies in **Table 4**.

**Table 4:** Additional Subgroup Analyses.

Sugroup Analysis	RR (95% CI)
*Country*	
United States, 13 studies	1.95 (1.28–2.97)*
Canada/Europe, 4 studies	2.25 (1.61–3.14)*
*Study Design*	
High Quality, 3 studies	3.22 (1.32–7.85)*
*Study Designed To Assess the Influence of Compensation*	
Dichotomous Data, 2 studies	1.74 (0.56–5.38)
Continuous Data, 4 studies	−8.05 (−11.08−5.03)*

* Demonstrates the negative influence of Workers’ Compensation.

## Discussion

This systematic review and meta-analysis demonstrates the negative influence that the presence of Workers’ Compensation has on patient outcomes following orthopaedic and trauma surgery. This comprehensive, best-evidence focused meta-analysis also showed consistent internal validity, reflecting our well-developed methodology, since the results are consistent in both overall and subgroup analyses. Our results demonstrate, in a simplified manner, that when a surgery is performed on a compensated patient with is known to be receiving compensation, surgeons should expect a 2-fold higher chance of obtaining an unsatisfactory outcome, when compared with non-compensated patients. The summary of this effect may used as a reference and taken into consideration for surgical decisions.

Studies have demonstrated that outcomes following orthopaedic surgery may be associated with factors other than compensation status. These studies suggest that this phenomenon may be influenced by biopsychological variables [57], [58], expectations or uncertainty regarding recovery [57], and low pain threshold [59]. However, though these associations are not strong [46].

In our protocol [31], we predicted that certain subgroups (e.g. non-traumatic spine or upper limb injuries) would demonstrate a higher risk of unsatisfactory results after surgery compared to the group as a whole. These hypotheses stemmed from reports that show a high prevalence of Workers’ Compensation patients within these populations[1], [5], [38], [55], [60]–[65]. Our results show no significant difference between these groups and our results showed a clear overlap of the confidence intervals between the overall and subgroup analyses. Two relevant issues should be raised to this point: 1) some subgroups may be more prone to selection bias; 2) certain subgroups may comprise of more complex patients who’s outcomes may be affected by other factors (as discussed above); and 3) we have made comparisons between patient cohorts that have been assessed using different criteria. This is was likely a considerable factor in the heterogeneity among our studies.

An additional subgroup analysis was performed, comparing cohorts grouped by country of origin, only high quality studies (judged by the risk of bias assessment tool) and studies specifically designed for assessing Workers’ Compensation cohorts. Only two of our included studies were specifically designed for assessing Workers’ Compensation and analysis did not show any differences between compensated and non-compensated cohorts. Since we have pooled only two studies for this particular analysis, we feel that this is underpowered.

It is important to compare the methodology of this systematic review with that of existing reviews in this area. In their review, Harris and colleagues [26] included all English studies in the existing literature relating to Workers’ Compensation and surgery outcomes. They included studies with both retrospective and prospective designs and did not seek to contact authors when additional data was needed from individual studies. Our approach differed in many aspects. For one, we only included prospective studies and studies that were published in the last 20 years. Secondly, we accounted for the quality of the included studies through use of an established tool. Thirdly, we performed several different subgroup analyses and lastly, we made attempts to contact the authors for missing data. We feel that by including non-prospective and older studies we would yield a biased magnitude of the risk, since these reports are related to a different employer-employee relation and retrospective designs may be prone to selection and measurement biases. Despite these methodological differences, our findings were similar. Harris reported an overall odds ratio of 3.79, (95% CI 3.28–4.37). Our results in the overall analysis can be converted to an odds ratio of 3.12 (95% CI 1.97–4.93). Despite of these overlapping confidence intervals, we feel that the results of Harris’ review maybe somewhat overestimated due to reporting bias.

The findings of this review have clear, direct implications on practice. The most explicit is the overall risk that is robust and homogeneous between the included subgroups. We also feel that the cohorts included in this systematic review are representative of the typical population of adults preparing for orthopaedic surgical intervention, which contributes to its high external validity. Also, because we specifically identified a subgroup of studies that focus on patient populations that are known to have a high prevalence of Workers’ Compensation cases, we can make the assumption that no subgroup of patients is more likely to be compensated in clinical practice. There is a less obvious message one can also gather from our review: the literature consistently demonstrated that compensated patients improve after surgical procedure, even if this improvement is delayed. Instead of avoiding surgical procedures for these populations, we recommend that surgeons take a patient-centred approach by balancing surgical indications and procedures with their own and the patient’ expectations.

From a practical perspective, we might state that our findings demonstrate with great external validity- that the surgical outcomes of Workers’ Compensation patients following orthopaedic surgery are somewhat worse than of similar patients who are not receiving compensation for their injuries. However, there is a notable lack of well-designed studies that explore the root causes of this clear association. This is an important consideration, given the current shift within surgery from a surgeon-centred responsibility to a circumstance-centred situation. A possible solution to this is to involve a multi-professional team in the management of Workers’ Compensation patients seeking care for their orthopaedic injuries. We also recommend that in addition to the standard medical history, physicians actively seek information to develop a comprehensive patient profile. Identifying potentially relevant social, economic, employment-related, and psychological issues may assist in establishing the prognosis following surgery and can aid in the decision-making process in practice regarding the patient’s treatment options.

We feel that we strengthened our review through inclusion of the rigorous quality assessment of the included studies. Our approach also recognized that no studies have previously reported sample size calculations for measuring this magnitude of the effect and as a way for establishing this cohort as representative of the population of interest. One possible sources of bias within our view is the fact that in most studies, the authors did not provide explicit criteria for what constitutes as a “Workers’ Compensation” patient. However, we feel confident in the definition of these patients, because there is little subjectivity in the assessment of how a patient is compensated.

A minor limitation that we encountered when conducting review was related to data abstraction. At times, the best available data may have been missed due to the inaccurateness of reporting and barriers to gathering data from the authors. Our experience showed that at least 70% of the unreported data was unavailable from the authors upon request. In this study, we experienced a 30% response rate after requesting unpublished data from authors, however only 1 of the 6 authors who responded was able to provide the data we requested.

This methodology and results of this study are quite comprehensive, robust and reproducible. Heterogeneity and publication bias may be a concern, but is inevitable when summarizing data from studies with different populations, measurements tools, and criteria. This is a recognized difficulty within orthopaedic research.

In future studies, comparisons between groups should be performed utilizing matched controls and should include statistical modifications to control group disparities, if any are present. In an ideal situation, matching should be introduced, which would eliminate the need for statistical adjustment. Another relevant factor that could be further explored is the standardizing surgical procedures and co-interventions (e.g. rehabilitation). Outcome assessors should be blinded to the patients’ compensation status and the patients should all be follow up for a similar time period. We feel that none of the studies included in this systematic review have reached these high methodological standards and we recommend that further research be initiated to resolve this relevant clinical question.

## Supporting Information

Appendix S1 **Search Strategy**
(DOC)

Appendix S2 **Quality Assessment Tool**
(DOC)
