# Correction: Workers’ Compensation Status: Does It Affect Orthopaedic Surgery Outcomes? A Meta-Analysis

**DOI:** 10.1371/annotation/17766d8c-c285-4e20-8cce-b2bed0be7c36

**Published:** 2013-08-02

**Authors:** Vinícius Ynoe de Moraes, Katelyn Godin, Marcel Jun Sugawara Tamaoki, Flávio Faloppa, Mohit Bhandari, João Carlos Belloti

The organization listed as providing the grant indicated in the Funding section is incorrect. The correct organization is: FAPESP (Fundação de Amparo à Pesquisa do Estado de São Paulo). 

