# Supplementary material for: Workers’ Compensation Status: Does It Affect Orthopaedic Surgery Outcomes? A Meta-Analysis
Source: PLoS One. 2012 Dec 5;7(12):e50251. doi: 10.1371/journal.pone.0050251 (PMC3515555; doi:10.1371/journal.pone.0050251)
Supplement: Appendix S1 — Search Strategy (DOC) [file pone.0050251.s001.doc]

**Appendix 1: Search Strategy**

Search Strategy: EMBASE (OVID) & MEDLINE (OIVD) -

--------------------------------------------------------------------------------

1 workers compensation.mp. or exp Workers' Compensation/

2 work$ compensation.mp. (7523)

3 1 or 2

4 exp Arthroplasty, Replacement/ or exp Orthopedic Procedures/ or exp Orthopedics/ or exp Pain, Postoperative/ or orthopaedic surgery.mp. or Fractures, Bone

5 3 and 4

***************************

Limits=from 1992 to 2012.

For the remaining databases: CINAHL, LILACS, Cochrane Library, Google Scholar

Free search for the words:

--------------------------------------------------------------------------------

1. workers compensation AND
2. orthopedic surgery

***************************

Limits=from 1992 to 2012.
