# Supplementary material for: Workers’ Compensation Status: Does It Affect Orthopaedic Surgery Outcomes? A Meta-Analysis
Source: PLoS One. 2012 Dec 5;7(12):e50251. doi: 10.1371/journal.pone.0050251 (PMC3515555; doi:10.1371/journal.pone.0050251)
Supplement: Appendix S2 — Quality Assessment Tool (DOC) [file pone.0050251.s002.doc]

**Appendix 2: Quality Assessment Tool**

Tool to Assess Risk of Bias in Cohort Studies

1. Was selection of exposed and non-exposed cohorts drawn from the same population?

Definitely yes Probably yes Probably no Definitely no

(low risk of bias) (high risk of bias)

Examples of low risk of bias: Exposed and unexposed drawn for same administrative data base of patients presenting at same points of care over the same time frame

Examples of high risk of bias: exposed and unexposed presenting to different points of care or over a different time frame

2. Can we be confident in the assessment of exposure?

Definitely yes Probably yes Probably no Definitely no

(low risk of bias) (high risk of bias)

Examples of low risk of bias: Secure record [e.g. surgical records, pharmacy records]; Repeated interview or other ascertainment asking about current use/exposure

Examples of higher risk of bias: Structured interview at a single point in time; Written self report; Individuals who are asked to retrospectively confirm their exposure status may be subject to recall bias – less likely to recall an exposure if they have not developed an adverse outcome, and more likely to recall an exposure (whether an exposure occurred or not) if they have developed an adverse outcome.

Examples of high risk of bias: uncertain how exposure information obtained

3. Can we be confident that the outcome of interest was not present at start of study

Definitely yes Probably yes Probably no Definitely no

(low risk of bias) (high risk of bias)

4. Did the study match exposed and unexposed for all variables that are associated with the outcome of interest or did the statistical analysis adjust for these prognostic variables?

Definitely yes Mostly yes Mostly no Definitely no

(low risk of bias) (high risk of bias)

Examples of low risk of bias: comprehensive matching or adjustment for all plausible prognostic variables

Examples of higher risk of bias: matching or adjustment for most plausible prognostic variables

Examples of high risk of bias: matching or adjustment for a minority of plausible prognostic variables, or no matching or adjustment at all. Statements of no differences between groups or that differences were not statistically significant are not sufficient for establishing comparability.

5. Can we be confident in the assessment of the presence or absence of prognostic factors?

Definitely yes Probably yes Probably no Definitely no

(low risk of bias) (high risk of bias)

Examples of low risk of bias: Interview of all participants; self-completed survey from all participants; review of charts with reproducibility demonstrated; from data base with documentation of accuracy of abstraction of prognostic data

Examples of higher risk of bias: Chart review without demonstration of reproducibility; data base with uncertain quality of abstraction of prognostic information

Examples of high risk of bias: Prognostic information from data base with no available documentation of quality of abstraction of prognostic variables

6. Can we be confident in the assessment of outcome?

Definitely yes Probably yes Probably no Definitely no

(low risk of bias) (high risk of bias)

Examples of low risk of bias: Independent blind assessment; Record linkage; For some outcomes (e.g. fractured hip), reference to the medical record is sufficient to satisfy the requirement for confirmation of the fracture.

Examples of higher risk of bias: Independent assessment unblinded; self-report; For some outcomes (e.g. vertebral fracture where reference to x-rays would be required) reference to the medical record would not be adequate outcomes.

Examples of high risk of bias: uncertain (no description)

7. Was the follow up of cohorts adequate?

Definitely yes Probably yes Probably no Definitely no

(low risk of bias) (high risk of bias)

Examples of low risk of bias: No missing outcome data; Reasons for missing outcome data unlikely to be related to true outcome (for survival data, censoring is unlikely to introduce bias); Missing outcome data balanced in numbers across intervention groups, with similar reasons for missing data across groups; For dichotomous outcome data, the proportion of missing outcomes compared with observed event risk is not enough to have a important impact on the intervention effect estimate; For continuous outcome data, plausible effect size (difference in means or standardized difference in means) among missing outcomes is not large enough to have an important impact on the observed effect size; Missing data have been imputed using appropriate methods.

Examples of high risk of bias: Reason for missing outcome data likely to be related to true outcome, with either imbalance in numbers or reasons for missing data across intervention groups; For dichotomous outcome data, the proportion of missing outcomes compared with observed event risk is enough to induce important bias in intervention effect estimate; For continuous outcome data, plausible effect size (difference in means or standardized difference in means) among missing outcomes is large enough to induce clinically relevant bias in the observed effect size; Potentially inappropriate application of simple imputation.

8. Were co-Interventions similar between groups?

Definitely yes Probably yes Probably no Definitely no

(low risk of bias) (high risk of bias)

Examples of low risk of bias: Most or all relevant co-interventions that might influence the outcome of interest are documented to be similar in the exposed and unexposed.

Examples of high risk of bias: Few or no relevant co-interventions that might influence the outcome of interest are documented to be similar in the exposed and unexposed.
